# Functional Neurological Symptom Disorder (FND) Leading to the Development of Deep Vein Thrombosis (DVT)

**DOI:** 10.7759/cureus.26378

**Published:** 2022-06-27

**Authors:** Ahmad Othman, Arthur Cecchini, Amira Eftaiha, Nneka Nwosisi, Deidre Pierce

**Affiliations:** 1 Internal Medicine, East Tennessee State University Quillen College of Medicine, Johnson City, USA; 2 Emergency Medicine, King Abdullah University Hospital, Irbid, JOR; 3 Medical Student, East Tennessee State University Quillen College of Medicine, Johnson City, USA

**Keywords:** deep vein thrombosis, physical therapy, psychiatry, internal medicine, functional neurological disorder

## Abstract

Functional neurological symptom disorder (FND) remains a clinical challenge. It is one of the many mimics of cerebrovascular accidents, spinal cord disorders, and lower motor neuron disease. Patients often undergo an extensive workup to exclude other causes of neurological dysfunction before the diagnosis is made. FND is often associated with weakness and paralysis, yet we could not locate a case depicting symptoms severe enough to cause venous thromboembolism. We present a patient diagnosed with FND who subsequently developed deep vein thromboses (DVT) of the bilateral lower extremities. She was placed on systemic anticoagulation and her functional symptoms improved with physical therapy (PT). This case describes the need for early PT to improve function and prevent complications related to functional immobility.

## Introduction

Functional neurologic symptom disorder (FND) comprises of neurologic symptoms causing significant functional impairment, and it carries a poor long-term prognosis [1]. The etiology of FND is unknown. Risk factors for FND include maladaptive personality traits, a history of abuse and neglect in childhood, and coexisting neurological disease. FND is often precipitated by recent psychological stress or the onset of another neurological illness [2-4]. The diagnostic criteria for FND include one or more symptoms of altered sensory or voluntary motor function, symptoms not better explained by another medical or mental disorder, clinical findings incompatible with any other known neurological or medical condition, and the symptom or deficit causes significant distress or impairment in function [2]. In patients with predominant motor involvement, weakness is the most common symptom. Tremor, jerking movements, dystonia, abnormal limb posturing, or gait abnormalities may also be present. Symptoms may be unilateral or bilateral [2,5-7]. Clinical examination findings consistent with FND include the Hoover sign, collapsing or give-way weakness, motor inconsistency, tremor entrainment, and hemifacial overactivity [2,8]. Treatment of FND includes patient education regarding the diagnosis and circumvention of maladaptive behavior. Physical therapy (PT) and cognitive behavioral therapy (CBT) are also recommended [9-11]. Physical-based therapies are especially helpful for improving functional motor symptoms. Other treatments described in the literature include botulinum toxin, therapeutic sedation, hypnosis, transcranial magnetic stimulation, and electromyographic feedback [7].

## Case presentation

A 55-year-old female presented to the hospital with minimal responsiveness, incomprehensible speech, and the inability to move her extremities. The history was obtained from family members who shared that she had felt weak, was having memory issues, and had progressive difficulty with ambulation for several weeks. The patient had also recently been under severe stress related to increasing difficulties taking care of her children with autism. The day before admission, the patient presented to an urgent care clinic due to nausea, vomiting, bilateral ear pain, and vertigo and she was prescribed meclizine and ondansetron. Emergency medical services (EMS) were called by her significant other on the day of admission, as she had worsening confusion, slurred speech, and minimal responsiveness.

On admission, vital signs revealed a temperature of 98.4°F, blood pressure of 120/64 mmHg, heart rate of 57 per minute, and oxygen saturation of 100%. The physical examination showed a lethargic patient with garbled and incomprehensible speech and an upper extremity tremor. She had a conjugate gaze and could follow a few basic commands, but she could not raise either her upper or lower extremities against gravity.

Complete blood counts (CBC) were unremarkable. A complete metabolic panel showed low potassium, elevated blood urea nitrogen (BUN), and metabolic acidosis. The urinalysis showed trace ketones and the urine drug screen (UDS) was negative. Her vitamin B12 level was borderline low, her vitamin D level was low, and C-reactive protein (CRP) was elevated (Table 1). The cerebrospinal fluid (CSF) analysis is shown (Table 2).

**Table 1:** Patient's initial laboratory results BUN: Blood urea nitrogen; HCO_3_: Serum bicarbonate; CRP: C-reactive protein

Laboratory studies	Patient values	Reference values
Leukocyte count (μL)	6300	3500-10,500
Hemoglobin (g/dL)	13.6	12.4-15.2
Platelet count (μL)	181,000	150,000-450,000
Sodium (mmol/L)	137	136-145
Potassium (mmol/L)	3.4	3.5-5.1
BUN (mg/dL)	21	6-20
Serum creatinine (mg/dL)	0.69	0.60-1.10
HCO_3_ (mmol/L)	18	22-32
Anion gap (mmol/L)	18	5-15
Vitamin B12 (pg/mL)	213	211-911
Vitamin D (ng/mL)	18	Deficient < 20. Insufficient 20 to <30. Toxicity > 150
CRP (mg/L)	101.7	0.0-10.0

**Table 2:** CSF analysis CSF: Cerebrospinal fluid

Laboratory studies	Patient values	Reference values
Color	Colorless	-
Appearance	Clear	-
Total nucleated cells (μL)	5	0-5
Red blood cells (μL)	2	0
Lymphocyte (%)	100	0-100
Glucose (mg/dL)	44	40-70
Protein (mg/dL)	33	15-45
Cryptococcal antigen	Negative	Negative
Culture	No growth	-
Gram stain	No white blood cells seen; no organisms seen	-

Computed tomography (CT) of the head without contrast, CT perfusion study, and CT angiography of the head and neck were unremarkable. Magnetic resonance imaging (MRI) of the brain revealed no intracranial abnormalities and an electroencephalogram (EEG) revealed no seizure activity. She was placed on intravenous thiamine, vitamin B 12 supplementation, vitamin D supplementation, intravenous fluids, and DVT prophylaxis with enoxaparin. Speech therapy and clinical nutrition consultations were obtained to optimize her oral intake. Serial physical examinations were performed (Table 3).

**Table 3:** Examination findings on first admission

Time and exam findings	Day one of the first admission	Day three of the first admission	Day of discharge during her first admission
Speech	Garbled and incomprehensible	Broken speech but comprehensible	Slow but comprehensible
Ability to follow commands	Able to smile, frown, and turn her head from side to side	Able to smile, frown, and nod	Able to follow commands
Right upper extremity strength	1/5	2/5	3/5
Left upper extremity strength	1/5	3/5	4/5
Right lower extremity strength	1/5	3/5	3/5
Left lower extremity strength	1/5	3/5	3/5
Tremor	Present	Not present	Present in left upper extremity yet decreased with distraction
Hand drop test	Positive	Positive	Positive
Give-away weakness	Unable to evaluate	Positive	Positive
Hoover sign	Unable to evaluate	Positive	Positive

During the initial hospitalization, she became more alert, began speaking, and she could move her extremities against gravity. More history was obtained from the patient, and she said she was previously admitted to an inpatient psychiatric service twice around 20 years ago due to separate suicide attempts, was previously on antidepressant medications, which she stopped taking several years ago, and previously required counseling due to post-traumatic stress disorder (PTSD), which was related to physical and emotional trauma during childhood. She denied any other past medical history.

Psychiatry and neurology consultations were obtained. After a group discussion of the patient’s past medical history, clinical presentation, and unrevealing workup, a diagnosis of functional neurologic symptom disorder was made. Psychiatry recommended outpatient follow-up for CBT, but the patient declined. Inpatient physical rehabilitation was also offered, but the patient declined. The patient began working with PT and was discharged home with home PT. Her CRP level decreased significantly by discharge (Table 4).

**Table 4:** CRP levels during first admission CRP: C-reactive protein

Test date	Day 1 of the first admission	Day 7 of the first admission	Reference range
CRP level (mg/L)	101.7	8.9	0-5

**Figure 1:**
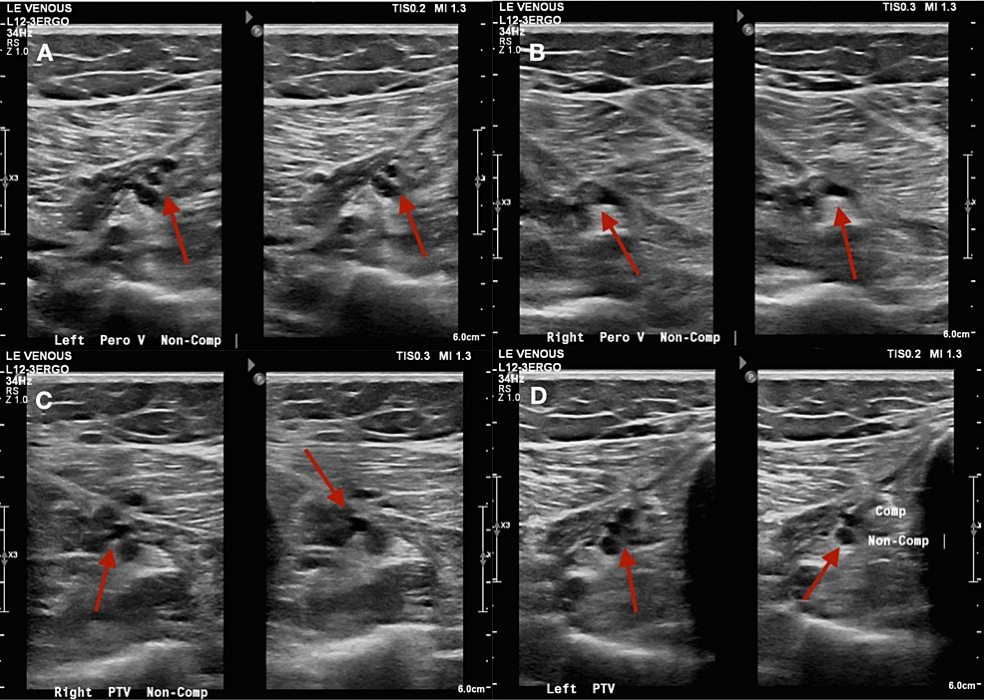
Venous doppler US showing bilateral DVT (A) Left peroneal vein (B) Right peroneal vein (C) Right posterior tibial vein (D) Left posterior tibial vein US: Ultrasound; DVT: Deep vein thrombosis

However, several days after discharge and before home PT started working with her, she was brought to the hospital again by her family because of worsening weakness and dysarthria. According to the family, the patient had new-onset bilateral lower limb pain and swelling. Doppler ultrasound (US) of the lower limbs was obtained revealing bilateral deep vein thromboses and the patient’s prophylactic dose of enoxaparin was changed to therapeutic dosing (Figure 1A-1D). 

**Figure 2:**
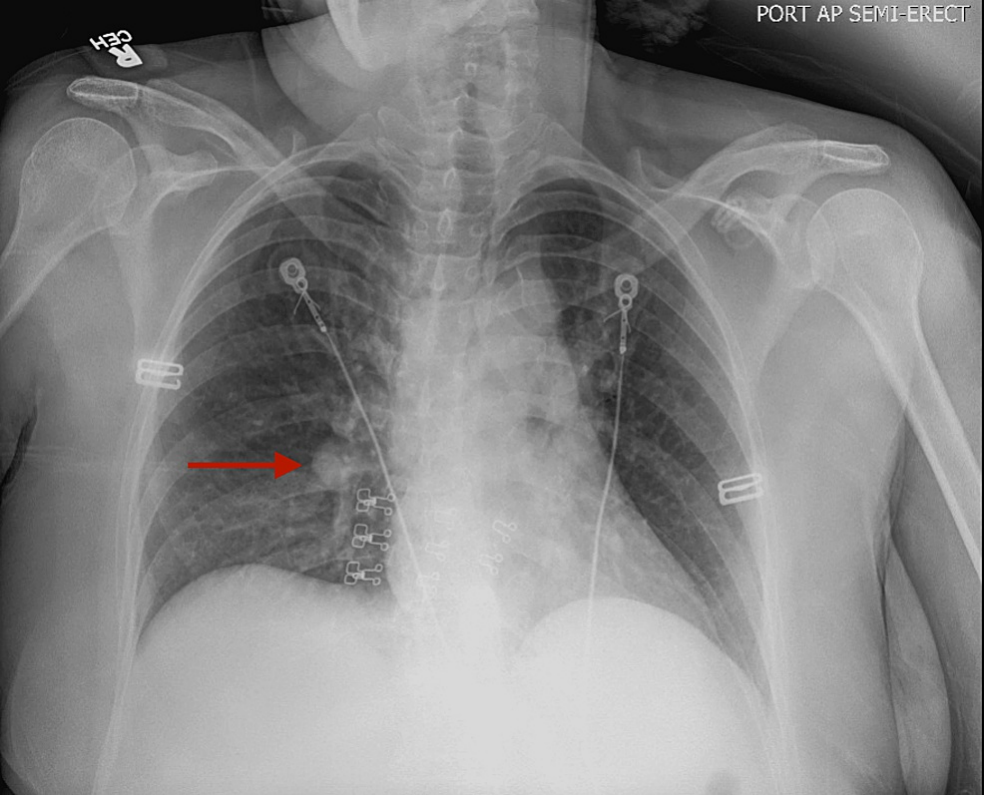
Plain radiography of the chest showing a 2 cm hilar nodule

**Figure 3:**
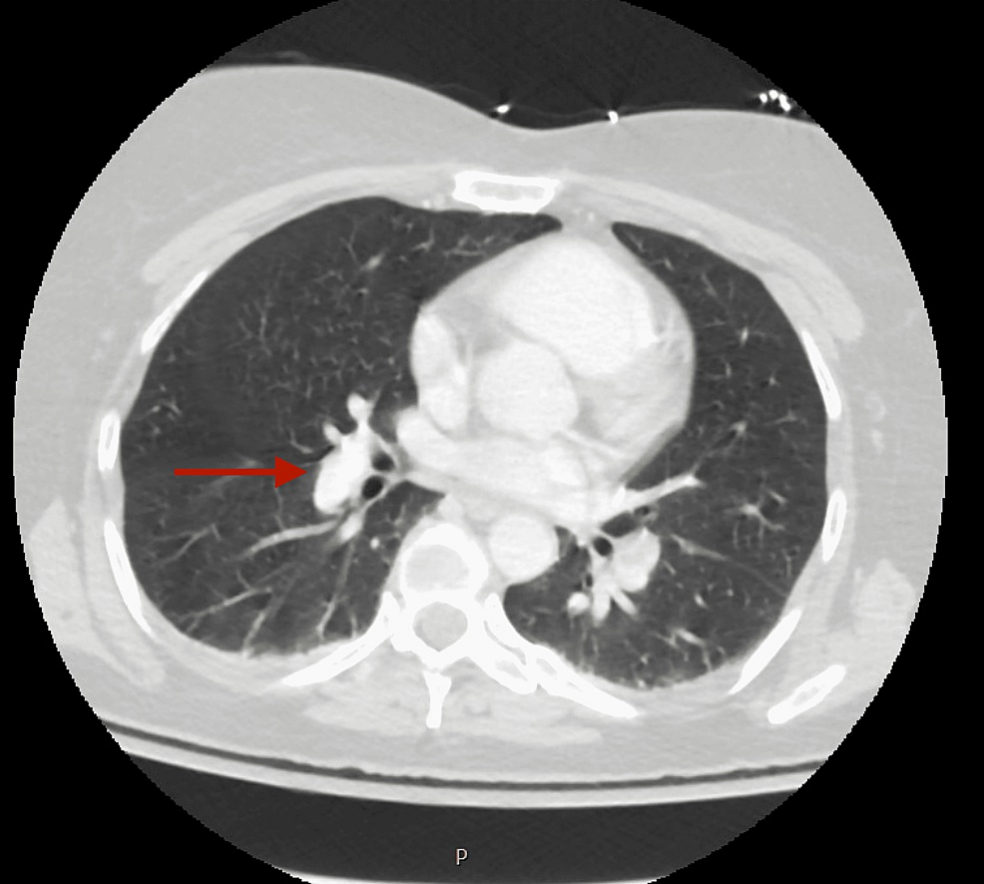
CT of chest with contrast showing the nodule discovered on plain radiography to be a pulmonary vessel CT: computed tomography

Further laboratory studies were performed including creatine kinase, antinuclear antibodies (ANA), folate levels, urine delta-aminolevulinic acid, and urine porphobilinogen, which all returned normal. MRI without and with contrast of the cervical, thoracic, and lumbar spine revealed no significant abnormalities. Chest radiography showed a 2 cm right hilar nodule and a follow-up CT of the chest revealed this to be a benign vessel (Figures 2, 3).

Serial physical exams were continued during the second admission, revealing a similar pattern seen during the previous hospitalization (Table 5).

**Table 5:** Examination findings on second admission

Time and exam findings	Day one of the second admission	Day five of the second admission	Day of discharge during her second admission
Speech	Garbled and incomprehensible	Slow, broken, but comprehensible	Comprehensible and fluid
Ability to follow commands	Unable to follow any commands	Able to smile, frown, and raise right upper extremity	Able to follow commands
Right upper extremity strength	2/5	3/5	4/5
Left upper extremity strength	1/5	1/5	3/5
Right lower extremity strength	2/5	2/5	3/5
Left lower extremity strength	1/5	1/5	3/5
Tremor	Not present	Present in left upper extremity only when asked if she has had tremor reoccurrence	Not present
Hand drop test	Positive	Positive	Positive
Give-away weakness	Unable to evaluate	Unable to evaluate	Positive
Hoover sign	Unable to evaluate	Unable to evaluate	Positive

Doppler US of the upper limbs was obtained as she described upper limb pain, but it was negative for DVT. Her CRP level was again found to be elevated on admission and nearly normalized later in the hospital course (Table 6).

**Table 6:** CRP levels during second hospitalization CRP: C-reactive protein

Test date	Day 1 of the second admission	Day 12 of the second admission	Reference range
CRP level (mg/L)	60.8	5.3	0-5

She was reevaluated by psychiatry and neurology, who supported her initial diagnosis of FND. She was started on venlafaxine and quetiapine. Mirtazapine was added later due to sleep disturbances and poor oral intake. Her condition started slowly improving, and she was discharged to an inpatient rehabilitation center.

## Discussion

FND is characterized by neurological symptoms that cause significant impairment in function but are inconsistent with a currently recognized neurological or psychological disease. The diagnosis of FND does not require the exclusion of feigned symptoms or attempts of secondary gain, as the motivation behind symptoms may be difficult to discern [2]. FND may cause severe physical disability equivalent to those with epilepsy or multiple sclerosis, is often associated with relapses, and carries a poor long-term prognosis [1,9]. The individual incidence of FND is difficult to calculate but is estimated to be 2-5/100,000 per year [2]. 

The pathogenesis and etiology of FND remain unclear, yet many factors are believed to play a role. Psychological factors such as trauma, conflicts, or life stressors may often, but not always, be associated with the onset of FND [2]. A meta-analysis showed that patients with FND are more likely to have experienced mistreatment or stressful life events, such as emotional neglect, physical abuse, or sexual abuse [3]. Some patients might have a preceding neurological illness, such as a cerebrovascular accident or onset of migraine disorder, before the onset of FND [4].

Unilateral weakness with or without paralysis is most common in patients with motor predominant FND, though paraparesis or tetraparesis may also occur [5,6,9]. Other motor symptoms include dystonia, abnormal posturing, tremor, jerking movements, and gait abnormalities [2,5,6]. Hoover sign, collapsing or give-way weakness, motor inconsistency, tremor entrainment, and hemifacial overactivity are often seen on physical examination [2,8].

The first-line treatment for FND is to explain and educate the patient about the diagnosis to circumvent maladaptive behavior. Physiotherapy and CBT are strongly recommended. Additional treatment options mentioned in the literature include botulinum toxin, therapeutic sedation, hypnosis, transcranial magnetic stimulation, and electromyographic feedback [7,10]. Pharmacologic therapy currently has no direct role in the treatment of FND, but it may be used to treat coexisting psychiatric or neurological disorders [9]. In patients with motor symptoms, PT has gained more traction in its effectiveness for patients with FND after the recent publication of several cohort studies and randomized controlled trials showing its benefit. Since it has been proposed that patient beliefs drive the pattern of movement in FND, the goal of PT is to retrain movement by refocusing attention and eliminating unhelpful disease beliefs and maladaptive behaviors [11]. Motor retraining involves the establishment of basic movement patterns, which gradually increase in complexity until normal movement patterns return. Distraction may also reduce symptom severity as a part of motor retraining [9,12].

This patient had a significantly elevated CRP for which we could not determine an organic cause. It is possible that the venous thrombosis caused the elevation, but it is mentioned in the literature that low-grade inflammation may be a mechanism for FND. We also noticed that her CRP levels tended to correlate with her levels of functional debility. One study has shown that in children and adolescents with FND, many but not all have elevations of the CRP level [13]. A strong potential association has not yet been shown in adult patients, but a potential correlation has been found in patients with predominantly motor symptoms, such as this patient [14,15]. Further studies would be beneficial to further delineate the relationship between CRP levels and FND.

## Conclusions

This patient presented with a severe case of FND subsequently leading to the development of acute lower limb DVT. She did not have any risk factors for the development of DVT except for her decreased mobility. We could not find any cases in the literature describing patients developing venous thrombosis related to debility caused by FND. Early mobilization in patients with FND is essential, even if therapy is limited to range of motion exercises to prevent complications, such as DVT. Further studies would be beneficial to determine the optimal modes of physical-based therapy in preventing complications and improving motor function in patients diagnosed with FND.
